# Effect of Poly(methacrylic acid) on the Cytokine Level in an In Vivo Tumor Model

**DOI:** 10.3390/molecules27144572

**Published:** 2022-07-18

**Authors:** Olga V. Zhukova, Evgenia V. Arkhipova, Tatiana F. Kovaleva, Daria A. Zykova, Natalya A. Dubovskaya

**Affiliations:** 1 Department of Pharmaceutical Chemistry and Pharmacognosy, Federal State Budgetary Educational Institution of Higher Education, Privolzhsky Research Medical University of the Ministry of Health of the Russian Federation, 603950 Nizhny Novgorod, Russia; zykovazda@gmail.com (D.A.Z.); nata.dubovskaya.99@bk.ru (N.A.D.); 2 Pre-Clinical Research Center, Centrak Research Laboratore, Federal State Budgetary Educational Institution of Higher Education, Privolzhsky Research Medical University of the Ministry of Health of the Russian Federation, 603950 Nizhny Novgorod, Russia; arhipova@nnovgorod.ru (E.V.A.); prazina@yandex.ru (T.F.K.); 3 Laboratory of Molecular Genetics, Center for Medical Genetics, University Clinic, Federal State Budgetary Educational Institution of Higher Education, Privolzhsky Research Medical University of the Ministry of Health of the Russian Federation, 603950 Nizhny Novgorod, Russia

**Keywords:** polymers of methacrylic acid, molecular-weight characteristics of polymer, immune system, interleukin, cytokine, cytokine level

## Abstract

Cancer is a leading cause of mortality globally. Despite remarkable improvements in cancer-treatment approaches, disease recurrence and progression remain major obstacles to therapy. While chemotherapy is still a first-line treatment for a variety of cancers, the focus has shifted to the development and application of new approaches to therapy. Nevertheless, the relationship between immune response, neoplastic diseases and treatment efficiency is not fully understood. Therefore, the aim of the study was to investigate the immunopharmacological effects of methacrylic acid homopolymer in an in vivo tumor model. Materials and methods: Monomeric methacrylic acid was used to synthesize polymers. Methacrylic acid was polymerized in dioxane in the presence of 4-Cyano-4-[(dodecylsulfanylthiocarbonyl)sulfanyl]pentanoic acid. To study the molecular weight characteristics of PMAA by GPC, carboxyl groups were preliminarily methylated with diazomethane. An experimental cancer model was obtained by grafting RMK1 breast cancer cells. The serum levels of IL-6, IL-10, IL-17, transforming growth factor β1 (TGF-β1), and tumor necrosis factor α (TNF-α) were measured by ELISA. Results: The effect of PMAA on the serum concentrations of several cytokines was studied upon its single administration to laboratory animals in early neoplastic process. The IL-6, IL-17 and TGF-β1 concentrations were found to change significantly and reach the level observed in intact rats. The IL-10 concentration tended to normalize. Conclusion: The positive results obtained are the basis for further studies on the effect of methacrylic-acid polymers with different molecular-weight characteristics on the neoplastic process.

## 1. Introduction

Cytokines play a leading role in regulating cell–cell interactions at the autocrine and paracrine levels and serve as the means of communication for innate and adaptive immune cells as well as nonimmune cells and tissues [1]. Cytokines are involved in the pathogenesis of tumor-related processes along with chemokines and growth factors [2]. The role of cytokines in the tumor process is impossible to determine unambiguously. On the one hand, the activation of angiogenesis, tumor progression and metastasis, as well as the immune evasion of cancer cells, are facilitated by cytokines. On the other hand, antitumor immunity is mediated by cytokines [3]. Moreover, cytokines mediate key interactions between immune and nonimmune cells in the tumor microenvironment (TME).

Such action of cytokines can be useful for improvement of anticancer therapies to promote effectiveness as well as to limit side effects [1].

Understanding this fact is of basic importance for developing new approaches to diagnosis and treatment of tumors (malignant neoplasms). Measuring the serum concentrations of the cytokines can be used to evaluate the immunity status and to monitor the disease [4,5]. 

Along with chemotherapy, immunotherapy has acquired special significance in treating oncology diseases [6]. Developing means to activate the anticancer immune protection mechanisms is a pressing problem [7].

Polymeric particles are of interest to study in this context because they are used as carriers to deliver anticancer agents. Synthetic polyelectrolytes are not antigenic per se. However, they act to enhance the immune response when administered together with antigens, thus acting as adjuvants. An advantage of this property is taken in vaccine design and development [8].

Therefore, the aim of the study was to investigate the immunopharmacological effects of methacrylic acid homopolymer in an in vivo tumor model. The levels of IL-6, IL-10, IL-17, transforming growth factor β1 (TGF-β1) and tumor necrosis factor α (TNF-α) in serum were measured to investigate the relationship between these indicators, cancer and treatment. 

## 2. Materials and Methods

### 2.1. Poly(methacrylic acid) (PMAA) Synthesis

Monomeric methacrylic acid (MAA) (Aldrich) was used to synthesize polymers. MAA was distilled at a lower pressure prior to use. The initiator azoisobutyric acid dinitrile (AAD) was recrystallized from methyl tert-butyl ether, vacuum-dried and stored at 0 °C; the purity was checked by ^1^H NMR spectroscopy. The ^1^H NMR spectrum (CDCl_3_, δ, ppm): 1.7 (s, 11H). 4-Cyano-4-[(dodecylsulfanylthiocarbonyl)sulfanyl]pentanoic acid (CDSPA, C_12_H_25_SC(=S)SC(CH_3_)(CN)CH2CH2COOH) (97%, TSI) was used as a reversible chain transfer (RCT) agent. The solvents dioxane, methanol, THF and DMSO were purified by conventional methods [9].

### 2.2. Polymerization

MAA was polymerized in dioxane (monomer:solvent = 2:1 (*v*/*v*)) in the presence of CDSPA in sealed ampoules at 70 °C. The mixture was preliminarily degassed via three freeze–pump–thaw cycles. MAA polymerization was carried out for 8 h. The initiator was used at 2 × 10^–3^ mol/L monomer. The RCT agent concentration was varied from 0.01 to 0.1 mol/L. The resulting polymers were precipitated with cold diethyl ether from ethanolic solutions three times to purify and dried to a constant weight in vacuum at room temperature.

### 2.3. Molecular-Weight Characteristics of Polymers

To study the molecular-weight characteristics of PMAA by GPC, carboxyl groups were preliminarily methylated with diazomethane. GPC was run on a Shimadzu Prominence LC–20VP chromatograph with Tosoh Bioscience columns filled with polystyrene gel (pore sizes 1 × 10^5^ and 1 × 10^4^ Å) at 40 °C. THF was used as an eluent; the eluent flow rate was 0.7 mL/min; a differential refractometer was used for detection. Chromatograms were processed using LCsolution software. Narrow-disperse PMAA standards were used for calibration.

### 2.4. Experimental Animals

Experiments were carried out in Wistar rats (females; weight: 260.0 ± 10 g; age at the beginning of the experiment: 3 months). Animal rearing at the certified breeding facility of the Central Research Laboratory of the Privolzhsky Research Medical University (RF Ministry of Health) complied with the Health and Hygiene Standards SP 2.2.1.3218-14. All experiments were performed in accordance with the Guide for the Care and Use of Laboratory Animals (National Research Council, 2011) and the European Convention for the Protection of Vertebrate Animals Used for Experimental and Other Scientific Purposes (Strasbourg, 2006) and were approved by the Ethics Committee at the Privolzhsky Research Medical University.

### 2.5. In Vivo Subcutaneous Cancer Model

An experimental cancer model was obtained by grafting RMK1 breast cancer cells, which were purchased from the Blokhin Oncology Research Center.

Transplantation began with anesthesia of the donor rat, then the subcutaneous tumor was cut out and crushed, as a result of which cancer cells were suspended in sterile Hanks’ solution at a ratio of 50 mg per 0.5 mL. Cell suspension was injected to the recipient rat subcutaneously into the armpit area axillary region. The day of transfusion of injection was taken as 0 days of tumor development. Test compounds were injected intraperitoneally with 9 mg/kg polymeric system once on the 10th day, which corresponded to the beginning of oncogenesis and formation of tumor nodes and activation of the immune system [10]. Control rats received PBS. On the 16th day, animals were decapitated under isoflurane anesthesia.

To assess the level of cytokines, blood was collected in a test tube, centrifuged, and serum was taken away.

To assess the effect of methacrylic-acid polymers on the immune system, the animals were divided into the following groups: healthy animals without tumors (Intact control) in the amount of 4; animals with RMK1 (Tumor control) in the amount of 4; animals with RMK1, which were administered the system, in the amount of 4.

### 2.6. Serum Cytokine Measurements in Tumor-Bearing Rats by ELISA

The serum levels of IL-6, IL-10, IL-17, transforming growth factor β1 (TGF-β1), and tumor necrosis factor α (TNF-α) were measured by ELISA, using Cloud-Clone kits (United States) and an Epoch spectrophotometer (BioTek, Winooski, VT, USA).

### 2.7. Statistical Analysis

The mean values (M) and standard deviations (SD) were calculated to express the data. Quantitative variables were described by median (Me) with interquartile range (25th percentile; 75th percentile) in the case of a non-normal distribution or the mean (M) and standard deviation (SD) if the distribution was normal. The Mann–Whitney test was used to assess the significance of differences between the two groups (*p* < 0.05 was considered statistically significant).

## 3. Results and Discussion

MAA polymers with regulated molecular-weight characteristics were obtained by RCT polymerization, which proceeds via an addition–fragmentation mechanism. The use of RCT agents substantially reduces the molecular weight of the resulting polymers as compared with classical radical polymerization and narrows their molecular-weight distribution (Table 1).

The RAFT-prepared PMAA polymer were characterized by ^1^H NMR techniques. CDSPA-terminated polymer ^1^H NMR (400 MHz, CDCl_3_) spectra shows the resonance peaks at δ (ppm) = 3.6 (s, –COO**H**), 3.3 (m, –C**H_2_**–COOH), 2.6 (m, –C**H_2_**–CH_2_–COOH), 2.4–2.5 (tr, –C**H_2_**–S–), 1.7 (m, –C**H_2_**–CH_2_–S–), 1.8 (s, –C(**CH_3_**)CN–), 1.3 (s, –(C**H_2_**)_10_–) and 0.8 (tr, –C**H_3_**).The effect of polymers on cytokine production was studied in experiments with a rat model of RMK1 breast cancer. PMAA (M_n_∙10^3^ 99.2 Da, M_w_/M_n_ 1.57) was used to determine how the polymers affect the cytokine concentrations in the blood in tumor-bearing rats.

IL-6 plays a substantial role in the pathogenesis of cancer and is known to induce the synthesis and secretion of acute-phase proteins [11]. It can affect all aspects of tumorigenesis process by regulating proliferation, apoptosis, metabolism, survival, angiogenesis and metastasis. IL-6 can also modulate a tumor therapeutic resistance [12].

The pro-oncogenic effects of IL-6 were demonstrated in various cancer types, including breast cancer [13,14,15].

Associations of circulating IL-6 with breast cancer risk, prognosis in advanced cancer and side effects are a matter of discussion. Targeting IL-6 is considered to be a promising anticancer treatment [16].

Direct treatment of breast cancer cells with IL-6 inhibits their proliferation, while high circulating IL-6 levels correlate with poor prognosis in breast cancer patients. An increase in serum IL-6 provides a biomarker of tumor load and metabolic disorders. The discrepancy reflects the diverse effect of IL-6 [17,18].

The serum IL-6 level was significantly increased in control tumor-bearing rats (5.13 ± 0.76 pg/mL) in comparison with intact animals (2.79 ± 0.76 pg/mL).Increased levels of IL-6 in the serum and tumor site were demonstrated in several cancers, including breast cancer [12].

Blocking IL-6 or inhibiting the IL-6 downstream signaling pathways was shown to provide therapeutic gain in cancers, which are associated with a higher level of IL-6 [19].

**Figure 1:**
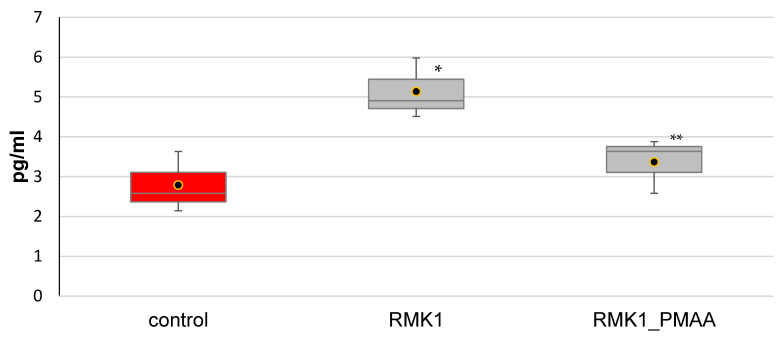
IL-6 concentration in the blood serum in rats administered with PMAA. (The difference from (*) intact rats or (**) control RMK1 tumor-bearing rats was significant at *p* < 0.05).

The serum IL-6 concentration was observed to significantly decrease after single PMAA administration in rats grafted with RMK1 cells (3.37 ± 0.69 pg/mL) as compared with control tumor-bearing rats (Figure 1). Downregulation of IL-6 might be related to the better response to treatment.

As for IL-10, impairment of the antitumor immune response in the tumor microenvironment to facilitate cancer immune evasion and stimulation of angiogenesis was most often reported as its pro-oncogenic effects [20]. Its anticancer effects include activation of natural killer cells and inhibition of reactive oxygen species [21,22].

**Figure 2:**
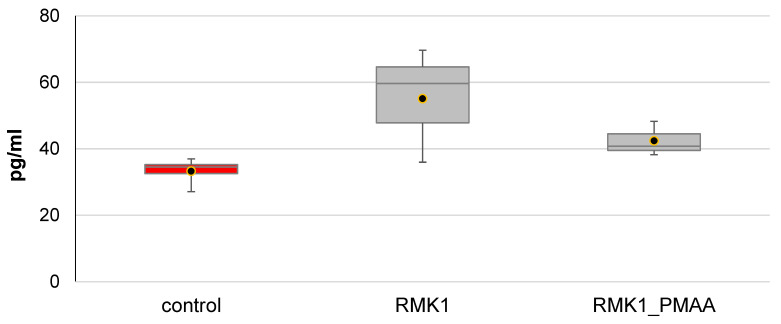
Changes in IL-10 concentration in response to PMAA administration.

The IL-10 concentration tended to decrease after PMAA administration to RMK1 tumor-bearing rats (42.40 ± 5.21 pg/mL) as compared with the control rats (55.08 ± 17.30 pg/mL), while no significant difference was observed in comparison with the intact rats (33.26 ± 4.27 pg/mL) (Figure 2).

Like many other cytokines, IL-17 plays a dual role in the neoplastic process [].Activation of angiogenesis is an established pro-oncogenic effect of IL-17. Its anticancer effects include stimulation of the antitumor cytotoxic T-cell response [23].

**Figure 3:**
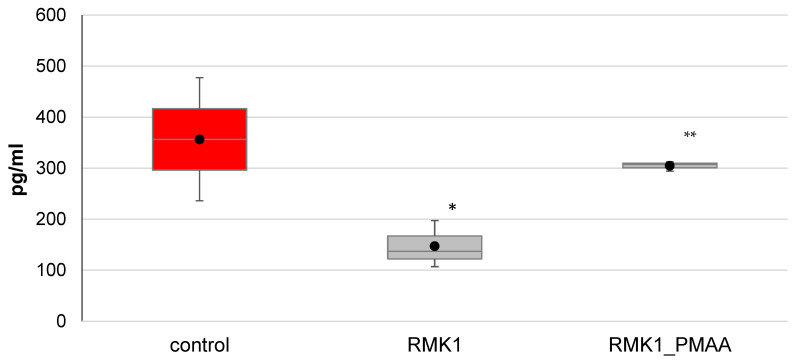
Changes in IL-17 concentration in response to PMAA administration. (The difference from (*) control rats or (**) control RMK1 tumor-bearing rats was significant at *p* < 0.05).

The IL-17 concentration significantly decreased in the early neoplastic process, from 356.54 ± 120.58 pg/mL in the intact rats to 147.14 ± 45.96 pg/mL in the rats grafted with cancer cells. Single administration of PMAA to tumor-bearing rats significantly increased the IL-17 concentration to 304.59 ± 9.62 pg/mL (Figure 3), suggesting a normalization of the parameter.

TGF-β1 is a regulatory cytokine that both exerts a suppressor effect and facilitates the neoplastic process in breast cancer cell lines and tissues [24]. A dual function is performed by TGF-β1 in tumor progression [25]. Acting as a tumor suppressor, TGF-β1 exerts an antiproliferative effect in early tumorigenesis. TGF-β1 inhibits cell-cycle progress and facilitates apoptosis, thus exerting a tumor-suppressing effect. Estrogen receptor-mediated proliferation is limited by TGF-β1 [26]. At the same time, cancer cells are capable of evading the suppressor effect of TGF-β1 at more advanced stages, leading to tumor progression [27,28]. TGF-β1 exerts a stimulatory effect on cancer cells to increase their invasion and metastasis [29].

In breast cancer, TGF-β enhances the vasculature within TME by regulating the expression of VEGF and MCP-1 [30].

Thus, the pleiotropic nature of TGF-β signaling was shown to be associated with drug resistance, tumor escape and the undermining of clinical response to therapy in a variety of cancers, including breast cancer [2,31].

Measurement of TGF-β pathway components in blood and tumor tissues represents a rapid and accurate approach to determine cancer risk, stratify patients into treatment populations and predict response to treatment. High levels of TGF-β in patients with breast cancer were shown to predict a poor prognosis. At the same time, TGF-β1 may be a serum predictor that becomes altered well before the development of clinically detectable tumors [26,32,33,34,35].

**Figure 4:**
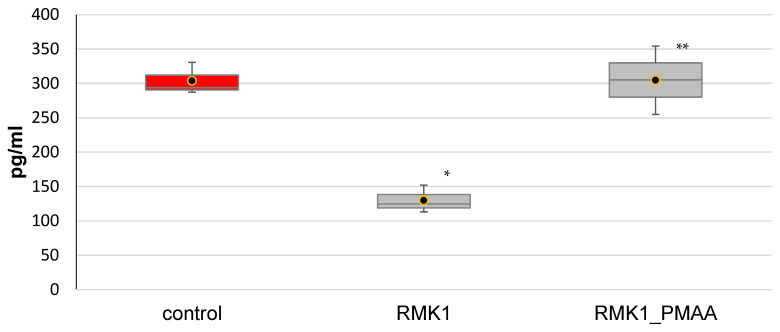
Changes in TGF-β1 concentration in response to PMAA administration. (The difference from (*) intact rats or (**) control RMK1 tumor-bearing rats was significant at *p* < 0.05).

In this study, TGF-β1 was found to significantly decrease in the early neoplastic process, from 303.80 ± 23.35 pg/mL in the intact rats to 129.94 ± 20.03 pg/mL in the rats grafted with cancer cells. PMAA normalized the TGF-β1 concentration to 303.76 ± 49.74 pg/mL (Figure 4).

TNF-αRMK1 is an inflammatory cytokine produced during acute inflammation and is responsible for a diverse range of signaling events within cells, leading to necrosis or apoptosis [36,37,38].

It plays an important role in tumor development as well [39]. The aberrant expression of TNF-α was found in a variety of tumors, including breast cancer [40]. The antitumor activities of TNF-α were used in cancer treatments [41]. TNF-α induces inflammation, leading to necrosis of tumor tissue. Blocking TNF-α activity was discovered to reduce the toxicity and may enhance the therapeutic effect of immune checkpoint inhibitors [42,43,44]. At the same time, the cytokine was found to show cancer-promoting effects [37].

TNF-α plays a critical role in tumor signaling pathways and immune-cell manipulation within the TME.TNF-α induces diverse effects that are both oncogenic and tumor-suppressive [45].

Thus, TNFα is a pleiotropic cytokine that can trigger opposing events in target cells, which differ in normal and malignant cells [46].

The understanding of cellular and molecular mechanisms of TNF pleiotropic effects might reveal novel drug targets for the treatment of cancer [47].

**Figure 5:**
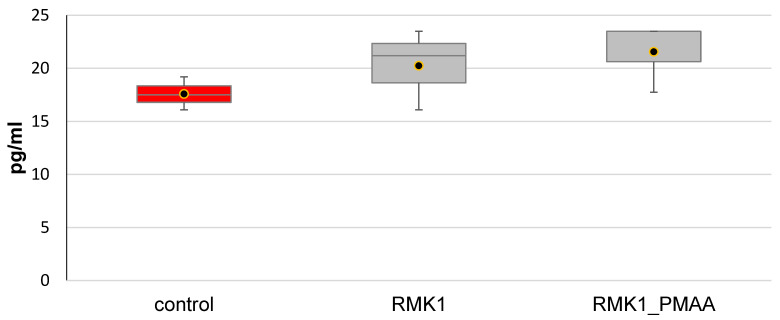
Changes in TNF-α concentration in response to PMAA administration.

In this study, the TNF-α concentration was also measured in our study and showed no change in the rats with a neoplastic process (20.26 ± 3.79 pg/mL) as compared with the intact rats (19.73 ± 4.46 pg/mL) or in response to PMAA administration (21.58 ± 3.32 pg/mL) (Figure 5). Such effects may depend on a premalignant/malignant condition of the cells.

It should be noted that PMAA exerts an antitumor effect [48]. PMAA is known as a potential carrier of anticancer drugs. In 2016, anticancer properties were studied both in vitro and in vivo for PMAA combined with gold-containing nanoparticles and conjugated to doxorubicin through an acid-labile cysteine bond [49]. A high efficacy of the conjugate in chemotherapy and radiotherapy was demonstrated with a human cervical adenocarcinoma cell line. 

A universal model of pH-sensitive nanoparticles designed for selective drug delivery was proposed in 2011, and PMAA was a component [50]. Mesoporous silica nanoparticles were coated with chitosan and PMAA. Doxorubicin was used as a model agent to study the nanoparticle behavior in conditions mimicking the biological media. The MTT cytotoxicity assay showed that empty carrier microspheres are suitable as a drug vehicle. A similar system was described in 2014 [51]. A high encapsulation efficiency towards doxorubicin was demonstrated again for nanoparticles. In 2015, Zhao, Yang and Li et al. described graphene-oxide nanoparticles modified with polyethylene glycol and PMAA [52]. The content of PMAA segments was 33 wt%. PMAA segments were found to substantially reduce premature doxorubicin release in stimulated normal tissues and to increase doxorubicin release in stimulated cancer tissues. Glutathione was used as a stimulating agent. The carriers showed a sixfold increase in release rate at pH 5.0 in the presence of 10 mM glutathione (stimulated cancer tissues) as compared with that at pH 7.4 in the presence of 10 mM glutathione (stimulated normal tissues). By an in vitro cytotoxicity assay, the carriers showed good cytocompatibility, and when loaded with doxorubicin, efficiently killed SiHa cervical cancer cells.

In 2012, PMAA nanogels were described as carriers of anticancer drugs [53]. PMAA fragments were linked together using N,N-bis(acryloyl)cystamine. The macromolecules break into oligomeric fragments with a molecular weight of approximately 1200 Da at these bonds when exposed to glutathione or dithiothreitol, which is used to induce endoplasmic reticulum stress. Compounds with this molecular weight are easily eliminated from the body. Doxorubicin was efficiently loaded in nanohydrogels (up to 42.3 wt%). Its complexation was due to strong electrostatic interactions between the amino group of doxorubicin and carboxyl groups of PMAA at physiological pH. The release rate and percent release of doxorubicin from doxorubicin-loaded nanohydrogels were low (<15 wt% within 24 h) at pH 7.4 and significantly higher (>91 wt% within 5 h) at lower pH 5.0 (in the presence of reducing agents).

A triple copolymer of starch, PMAA, and polysorbate 80 has been described as a carrier for doxorubicin loading and subsequent pH-dependent release [54,55]. The resulting doxorubicin-loaded nanoparticles were proposed as a means to overcome multidrug resistance in human breast cancer cells. The systems were tested in vitro. The doxorubicin content in the nanoparticles was 49.7 ± 0.3%. Doxorubicin was released more readily and faster at acidic pH because its molecular interactions with the polymer were weaker in an acidic milieu. Cytotoxicity testing in MDR1 cells showed that a 20-fold decrease in IC50 was achieved with doxorubicin-loaded nanoparticles compared with pure doxorubicin, suggesting high selective cytotoxicity for the nanoparticles.

However, the effect of PMAA on the neoplastic process has not been evaluated as of yet. This circumstance determines the importance of our study.

There is evidence that polyanions have antitumor activity, which is manifested in vivo and is associated with macrophage activation (Breing M.C., Munson A.E., Morahan P.S., 1978).

A range of evidence suggests a complex system of natural and acquired antitumor defense of the body, and increasing importance in this system is given to macrophages.

The action of polymeric anions is based on mechanisms related to the macromolecular nature. One of these properties is the ability for multipoint cooperative interaction with other chemically complementary macromolecules to form stable interpolymer complexes or strong multipoint cooperative adsorption on chemically complementary surfaces. Linear polyelectrolytes are able to glue B-lymphocytes to T-helper cells due to multipoint adsorption of linear macromolecules on cell membranes. The macrophage can also participate in agglomeration. Synthetic polyelectrolytes enhance the effect of T-B cooperation. Thus, the immunostimulatory and antitumor activity of polyanions is associated with their direct effect on macrophages and with the ability to activate them [56].

However, there was no assessment of the independent influence of PMAA on the tumor process, or on the state of immune system in the development of the tumor process. This fact defines the prospects of the presented research.

Earlier we proposed a scheme of the influence of IL-10 and IL-17 on the tumor process [57]. IL-10 promotes conversion of M1 macrophages (classically activated, with phagocytosis as a main function) to M2 macrophages (alternatively activated macrophages, TAMs, which facilitate tumor cell evasion from the immune system) [58]. Moreover, M2 macrophages are known to express ample IL-10 receptors and to secrete IL-10 [59]. As a proinflammatory cytokine, IL-10 stimulates the suppressor cells, the main function of which is to inhibit secretion of cytokines, including IL-17. Stimulation of the antitumor cytotoxic T-cell response is thus suppressed, and the tumor process spreads [60]. This information is included in the Discussion section.

## 4. Conclusions

A dual role, which is associated with the stimulatory and inhibitory effects on the tumor, is played by cytokines in the majority of cases.

The effect of PMAA on the serum concentrations of several cytokines was studied upon its single administration to laboratory animals in early neoplastic process. The IL-6, IL-17 and TGF-β1 concentrations were found to change significantly and reach the level observed in intact rats. The IL-10 concentration tended to normalize. The data obtained suggest that PMAA has a positive effect on the neoplastic process at an early stage of its development. The results obtained form the basis for further research into the evaluation of PMAA of different molecular weights on different tumor-development processes.

## Figures and Tables

**Table 1:** Molecular-weight characteristics of PMAA synthesized in the presence of CDSPA, [AAD] = 0.002 mol/L, T = 70 °C.

[RCT Agent], mol/L	Characteristics		
	M_n_∙10^3^	M_w_∙10^3^	M_w_/M_n_
0.01	99.2	160.4	1.57
0.04	31.5	40.0	1.27
0.08	19.5	23.6	1.19
0.10	14.6	16.6	1.13

## Data Availability

The data presented in this study are available on request from the corresponding author.
